# Big data analysis: examination of the relationship between candidates’ sociodemographic characteristics and performance in the UK’s Membership of the Royal College of Physicians Part 1 examination

**DOI:** 10.1007/s10459-024-10406-3

**Published:** 2024-12-20

**Authors:** Peter W. Johnston, Rute Vieira, Isobel M. Cameron, Ben Kumwenda, Kim A. Walker, Jennifer A. Cleland

**Affiliations:** 1 NHS Education for Scotland, Edinburgh, UK; 2 Centre for Healthcare Education Research and Innovation (CHERI), School of Medicine, Medical Sciences and Nutrition, University of Aberdeen, Room 2:040, Polwarth Building, Foresterhill, Aberdeen, AB25 2ZD UK; 3 Institute of Applied Health Sciences, School of Medicine, Medical Sciences and Nutrition, University of Aberdeen, Aberdeen, UK; 4 Centre for Medical Education, University of Dundee, Dundee, UK; 5 Lee Kong Chian School of Medicine, Nanyang Technological University Singapore, Singapore, Singapore

**Keywords:** Big data, Differential attainment, Performance, Postgraduate medical education, MRCP

## Abstract

Big datasets and data analytics enable granular analyses examining group differences in performance. Our focus is on differential attainment (DA) in postgraduate College (Board) examinations. We asked: Are candidates’ sociodemographic characteristics associated with performance on the UK’s Membership of the Royal College of Physicians (MRCP) Part 1 after adjusting for medical school performance (MSP) and type of medical programme? This was a retrospective longitudinal cohort study of 6040 medical graduates with linked sociodemographic data in the UK Medical Education Database qualifying from a UK medical school (2012–2014) and sitting MRCP Part 1 before October 2019. Chi-squared tests established univariable associations with MRCP performance (pass/fail first sitting MRCP Part 1). Multivariable mixed-effects logistic regression identified independent explanatory factors of success, adjusted for medical school. The odds (95% CI) of passing MRCP Part 1 exams on first sitting were greater for men (OR = 1.61, CI 1.42–1.81, *p* < 0.001) and those on a graduate entry programme (OR = 1.44, 1.05–1.99, *p* < 0.001). The odds of passing were lower as age increases (OR = 0.87, 0.85–0.90, *p* < 0.001), for minority ethnic (OR = 0.61, CI 0.53–0.7, *p* < 0.001), and gateway to medicine (OR = 0.49, CI 0.27–0.90, *p* = 0.02) candidates. After adjusting for MSP, odds were greater for passing in men (OR = 1.62, CI 1.24–2.11, *p* < 0.001) and candidates with higher MSP (OR = 4.12, CI 3.40–4.96, *p* < 0.001). Our findings illustrate how performance on MRCP part 1 is associated with group-level social and educational factors. This DA may be due to aspects of the assessment itself, and/or the persistent nature of social and educational disadvantage.

## Introduction

Candidate ability should determine success in exams, yet globally and at all levels of education there is much evidence of variation in performance on educational assessments between learners from different societal groups (Arday & Mirza, 2018; Li, 2018; Smith, 2020). Differential Attainment (DA) or group-level differences in performance of groups (rather than individual differences) is also observed across all medical education stages and medical specialties.

Big datasets and big data analytics have facilitated research examining DA patterns. Differential attainment in medical education is evidenced across various sociodemographic characteristics such as ethnicity (and race) (Stegers-Jager et al., 2012, 2015; Wijesekera et al., 2019; Wikaire et al., 2017; Woolf et al., 2011), age (Stegers-Jager et al., 2015; Wijesekera et al., 2019), gender (Haq et al., 2005; King et al., 2018; Stegers-Jager et al., 2015) and disability (Asghar et al., 2019; Hutchinson et al., 2021). Differential attainment is observed across multiple types of outcomes, including, but not limited to, clinical assessments (Stegers-Jager et al., 2012; Woolf et al., 2011), timely course completion (Stegers-Jager et al., 2015), speed of progression through the medical training pathway (Cleland et al., 2019), clerkship grades (Lee et al., 2007), in-course grade point average (Wikaire et al., 2017), multiple-choice exams (Hope et al., 2021; Woolf et al., 2011) and honour society inductions (Boatright et al., 2017; Wijesekera et al., 2019). Differential attainment in medical education and training can hinder individuals’ learning experiences and career progression (Ellis et al., 2022a; Kumwenda et al., 2018a, 2019; Teherani et al., 2018); limit diversity of the health workforce (Cantor et al., 1996; Rabinowitz et al., 2000) and ultimately impact negatively on patient care (Shen et al., 2018).

These last points are important: attracting future doctors from a range of backgrounds is critical in terms of ensuring fairness, supporting diversity and inclusivity, and in terms of patient care (Gomez & Bernet, 2019; LaVeist & Pierre, 2014). In many countries there is a policy mandate to widen access to medical school and to ultimately increase the diversity of the medical workforce, as well as an ethical and legal imperative to address inequalities in medical education and training (General Medical Council, 2018; Gonzaga et al., 2020). For example, in the UK, public authorities such as universities, Royal Colleges and the National Health Service (NHS) have a legal duty to address differences between groups with and without certain protected characteristics. Regulators and examination boards are now providing guidance on, for example, fair selection and assessment, and behaviour in the workplace.

Addressing the issue—and thus enabling equity and fairness within medical education and training—depends on identifying barriers to progression. A first step in doing this is to analyse routinely collected data to understand patterns of performance. These findings can then be used to provide guidance to operationalize and enact change including, for example, changing assessments so they do not discriminate against specific groups and/or providing appropriately targeted support. More broadly, issues identified in terms of examination performance can also be used to consider, and address, systemic and structural barriers to progression for certain groups in medical training (e.g., Orom et al., 2013; Woolf et al., 2016; Pyne and Ben-Schlomo, 2015).

Progression in data capture and linkage also allow the findings of earlier studies to be re-examined and extended. This too is important; medical education structures change over time and medical students themselves are changing, albeit slowly, in terms of sociodemographic variables (General Medical Council, 2019; Patterson, & Price, 2017). Patterns and associations seen in the past may be less relevant, and even misleading, today. Contemporary ‘big (education) data” characterised by linked datasets enables granular analyses to inform change.

Our focus in this paper is DA in postgraduate medicine College (Board) examinations. These examinations have a “gatekeeper” function: performance has a significant impact on the career progression of trainees (residents). Successful completion of these examinations is required to progress through training towards consultant status. For example, in the UK, successful completion of all three components of the Membership of the Royal College of Physicians examination (MRCP (UK) Part 1; MRCP (UK) Part 2 Written; and MRCP (UK) Part 2 Practical Assessment of Clinical Examination Skills (PACES)) is a prerequisite for internal medicine (previously core medical) training (e.g., cardiology, gastro-enterology).

While much work has examined the associations between candidate’s sociodemographic variables and progression through surgical training (e.g., Ellis et al., 2021; Ellis et al., 2022a; Scrimgeour et al., 2018a; Scrimgeour et al., 2018b, and see also Jones et al., 2024, for a recent review of the global literature on this topic), there is relatively little multi-site research on DA in physician/internal medicine training. Single-site and small multi-site studies are useful but not generalisable as different places may attract different trainees/residents. Moreover, this is an obvious gap in the literature given more candidates globally take physician/internal medicine postgraduate examinations than surgical ones. For example, in respect of the MRCPUK, taken by candidates from over 40 different countries, earlier studies indicated DA in respect of gender, ethnicity and/or country of origin in the MRCP Part 1 (Dewhurst et al., 2007; McManus & Wakeford, 2014; McManus et al., 2008; Wakeford et al., 2015) and differences in pass rates in graduates from different medical schools (McManus et al., 2008). While informative, these studies included a limited number of factors related with performance because of the datasets available at that time.

In this study, we used UKMED (Dowell et al., 2018) (https://www.ukmed.ac.uk/), a database which holds educational outcomes for all medical students and postgraduate trainees in approved UK medical schools and training programmes by crosslinking data from several sources, including the Higher Education Statistics Authority (HESA) Limited and the General Medical Council (GMC), to answer the research question: Do students’ sociodemographic characteristics influence performance in the MRCP Part 1 examination after adjusting for prior attainment at medical school and type of medical programme? We adjusted for prior academic attainment (candidates’ performance at medical school and on entry to the first step in UK medical postgraduate training) because previous research indicates a clear relationship between prior academic attainment at medical school and later performance (Ellis et al., 2022b; McManus et al., 2013; Paton et al., 2022; Scrimgeour et al., 2018a, 2018b).

## Methods

### Design

This was a retrospective longitudinal cohort study using data held in UKMED (https://www.ukmed.ac). Anonymised data were extracted for doctors sitting MRCP Part 1 who gained their primary medical qualification (PMQ) from a UK medical school in 2012, 2013 or 2014 and completing the United Kingdom Foundation Programme (UKFP) in 2014, 2015 and 2016 respectively. Data from these cohorts were available up to Autumn 2019.

### Context

It is useful here to give some context about the early stages of the UK’s medical education and training pathway. In the UK, most medical schools offer 5- or 6-year undergraduate programmes (the standard). There are specific 4-year graduate entry programmes although many medical students who decide to enter medicine as graduates choose instead to study standard length programmes. Gateway courses, or standard courses offering a  + 1 preliminary or gateway year, designed to attract students under-represented in medicine, are becoming an increasingly popular widening participation (WP) route into medicine in the UK. Their aims are to provide entry to medical schools, not normally accessible without the highest grades in secondary education, for students with educational and social disadvantage and to support these students to succeed (Medical Schools Council (2024). We were particularly interested in the associations between type of medical programme and later performance because, although gateway and graduate entry programmes were introduced in the UK to support widening access to medicine (Medical Workforce Standing Advisory Committee, 1997), evidence suggests that these students may experience continued disadvantage throughout medical education and training (e.g., Kumwenda et al., 2018a, 2019).

After medical school, graduates work as interns in the two-year Foundation Programme (UKFPO), successful completion of which allows them to apply for specialty training. In 2012–2014, medical school graduates/applicants to the UKFPO ranked their choice of the 21 foundation schools in order of preference (1–21), and allocation to Foundation training (offers) was based on an algorithm of the Foundation Training application score. For the data we accessed this application score was the sum of overall medical school performance (EPM) and performance on a Situational Judgement Test (SJT) designed specifically for selection into this first stage of postgraduate training (see later).

The EPM is the sum of three scores; points awarded for additional degrees (maximum of 5 points), points awarded for publications (maximum of 2 points) and a decile score based on their performance throughout medical school, ranging from 34 points for the 10th (lowest) decile to 43 points for students in the 1st (highest) decile. The Foundation Programme SJT is a written examination that aims to test the behaviours, traits and attitudes expected of doctors by the UK’s General Medical Council (Patterson et al., 2015). A combined score of the EPM and SJT was used to rank each graduate nationally for allocation to FP Training posts between 2013and 2024.

### Comparisons

We focused on examining differences between groups in terms of sex, ethnicity, age and socioeconomic status. Thus, and informed by previous UKMED studies examining differences in UK medical students and graduates (see https://www.ukmed.ac.uk/published_research), the following standardised and anonymised data were extracted from UKMED prior to analysis: sex (men, women); ethnicity (majority ethnic [White], minority ethnic [Asian/Asian British, Black/Black British, Mixed, Other Ethnic Groups]); age in years (at first MRCP Part 1 sitting); high school type (fee paying or state: (Kumwenda et al., 2017)); graduate status at the time of entry to medical school (graduate or non-graduate): (Kumwenda et al., 2018b); type of medical programme (standard entry, graduate entry, with preliminary year, with gateway year).

Socio-economic status is indicated in UKMED by several variables (e.g. parental occupation, first in family to attend higher education, family income). Previous analyses have highlighted that there is much collinearity between different socioeconomic indicators. We used parental occupation because it is strongly related to income and reflects social standing and social networks (Galobardes et al., 2006; Hosmer & Lemeshow, 2000). These variables were considered and categorised, following the *National Statistics Socio-economic Classification* (NS-SEC), which allowed us to extract and categorise parental occupation (professional/managerial versus non-professional/managerial occupation [Semi routine/routine, Lower supervisor/tech, Small employer/own, Intermediate]). Although many non-UK educated doctors sit the MRCP, the focus of our study was UK graduates only because sociodemographic indicators are context specific.

We also used EPM and SJT scores (see Context section earlier). Previous studies have found that the EPM decile score demonstrates the component most associated with prior attainment of the three component parts of the EPM. Therefore, and as per previous studies (Kumwenda et al., 2017, 2018a), EPM decile scores (not EPM total score) were used as a measure of prior academic attainment along with SJT score.

In respect of the outcome variable used to quantify performance at the MRCP exam, first attempt of MRCP Part 1 examination scores were used throughout, as these have been shown to be the best explanatory factor of future performance in postgraduate examinations (McManus & Ludka, 2012; Scrimgeour et al., 2017).

### Data management

All the requested data files were imported and merged using a combination of SQL server relational database management system (via UKMED) and within SPSS in a Data Safe Haven (Health Informatics Centre, University of Dundee). As such, these data were stored, handled, and analysed in accordance with high standards of security, governance, and confidentiality. At the time of carrying out this work, UKMED had ethics exemption for projects using exclusively UKMED data on behalf of all UK medical schools (https://www.ukmed.ac.uk/documents/UKMED_research_projects_ethics_exemption.pdf).

EPM data were ranked differently across the three cohorts. In 2012, graduating students were competition-ranked into quartiles within individual medical schools. The EPM ranking for the 2013 and 2014 cohorts was in deciles. Data provided as quartiles and deciles were transformed to mean score deviates following established methods (Garrud & McManus, 2018).

The analyses pertain to candidates from thirty medical schools. Medical schools are represented by a unique, anonymous identifier number. To control for the effect of students being clustered by medical school, we used HESA-records of the last medical school at which the student attended. Data from graduates from two medical schools—Cardiff and Swansea—were amalgamated as the University of Wales medical school as Swansea did not become an independent medical school until 2014.

HESA standard rounding methodology has been applied so that counts of people are rounded to the nearest of 5. Percentages are not provided where they are fractions of a group of people that is fewer than 22.5. There are also no averages presented where there are a group of seven or fewer people (*Rounding and suppression to anonymise statistics|HESA*). All models were fitted using complete data. The percentage of missing data was negligible for all variables except EPM and SJT.

### Data analysis

Data were analysed within the safe haven using IBM SPSS Statistics for Windows Version 27 (IBM Corp, Armonk, New York, USA). Descriptive statistics were produced. Categorical variables were summarised in counts and percentages. Continuous variables were summarised as means and standard deviations or as medians and interquartile ranges (as appropriate according to their distribution). Univariable analyses were conducted to assess the unadjusted relationship between the variables of interest and passing the first sitting of the MRCP Part 1. T-test was applied to compare means for normally distributed continuous variables, Mann–Whitney U test for non-normally distributed continuous variables and Chi-squared test were conducted to compare proportions of categorical variables. When assumptions were not met in the latter, the Fisher Exact test was applied instead.

A multivariable mixed effect logistic regression model, with medical school as a random effect, was applied, enabling us to adjust for the fact that students are clustered within medical schools. The dependent variable was pass/fail at MRCP Part 1. When deciding which variables to include in the models the following factors were considered: the results from the univariable analyses, hypotheses derived from earlier literature and the completeness of the data.

Two models were fitted. Model 1 was not adjusted for EPM and SJT scores since this would have resulted in a significant loss of data due to incomplete recording related to the later start of data collection for these variables in UKMED. However, to investigate the effect of these two variables on passing rate, these were included in Model 2.

The presence of multicollinearity was evaluated using variance inflation factors (VIF) (Marcoulides & Raykov, 2019). If the VIF was greater than 10, the variable would be removed from the model; if between 5 and 10, items were to be investigated and potentially excluded.

Effect sizes are reported as odds ratios and 95% confidence intervals.

## Results

Data were available for 6040 graduates with primary medical qualification from a UK medical school (2012–2014) sitting MRCP Part 1 before October 2019 (2012 n = 1905; 2013 n = 2085; 2014 n = 2050).

The results of univariable analyses between sociodemographic variables and MRCP Part 1 first attempt pass are shown in Table 1. We found statistically significant associations between MRCP Part 1 first sitting pass rates and age, sex, ethnicity, graduate status (but see later), parental occupation and school type (all *p* < 0.01).

**Table 1:** Passing the first part exam for MRCP (home students graduating in 2012, 2013, 2014) in relation to sociodemographic variables and prior educational attainment (EPM and SJT)

Characteristic	All	Pass stage 1	Fail stage 1	Significance
Median age (IQR)	N = 6040 26 (26, 28)	N = 3925 26 (26, 28)	N = 2115 27 (26, 28)	Mann–whitney U test *p* < **0.01**
Sex N (%) Women Men	N = 6040 3300 (55) 2740 (45)	N = 3925 2010 (61) 1920 (70)	N = 2115 1295 (39) 820 (30)	Chi square test *p* < **0.01**
Ethnicity N (%) Majority Ethnic (White) Minority Ethnic	N = 5745 4160 (72) 1585(28)	N = 3735 2780 (67) 955 (33)	N = 2010 1380 (33) 635 (67)	Chi square test *p* < **0.01**
Parent occupation N (%) Managerial/prof Non Managerial/prof	N = 5700 4305 (76) 1400 (24)	N = 3715 2850 (66) 870 (34)	N = 1985 1455 (34) 34 (66)	Chi square test *p* = **0.01**
High school type N (%) State Fee paying	N = 5985 4130 (69) 1855 (31)	N = 3900 2625 (64) 1275 (69)	N = 2085 1500 (36) 580 (31)	Chi square test *p* < **0.01**
Graduate on entry N (%) Graduate Non graduate	N = 6030 985 (16) 5045 (84)	N = 3920 585 (59) 3335 (66)	N = 2110 400 (41) 1710 (34)	Chi square test *p* < **0.01**
Course type N (%) Standard entry Graduate entry With prelim year With gateway year	N = 6025 5285 (88) 640 (11) 32 (1) 65 (1)	N = 3925 3465 (66) 420 (65) ^b^ 25 (39)	N = 2100 1825 (34) 225 (35) ^b^ 40 (61)	Chi square test *p* < **0.01**
Mean SJT equated (sd)	N = 1655 673.21 (25.6)	N = 935 676.61 (25.23)	N = 720 668.83 (25.46)	Mean diff = 7.78 95% CI (5.31, 10.24) *p* < **0.01**
Median EPM^a^ (IQR)	N = 5825 0.299 (− 0.376, 0.655)	N = 3750 0.299 (− 0.123, 1.00)	N = 2075 − 0.299 (− 1.01, 0.299)	Mann–whitney U test *p* < **0.01**

Statistically significant differences are shown in bold

^a^Normal deviate score

^b^N < 22.5 so not reported as per HESA guidelines

Those who passed MRCP Part 1 at first sitting were younger than those who did not [median 26 (IQR 26, 28) vs median 27 (IQR 26, 28)]. The pass rate for men (n = 1920, 70%) was significantly higher than for women (n = 2010, 61%). Candidates with a majority ethnic (white) background (n = 2780, 67%) had a significantly higher pass rate than those from minority ethnic background (n = 955, 33%). Candidates with parents with managerial/professional occupations were significantly more likely to pass (n = 2850, 66%) than those candidates whose parents had non managerial/professional occupations (n = 870, 34%). Candidates who had attended state schools (n = 2625, 64%) were significantly less likely to pass MRCP Part 1 on first sitting than those from fee paying schools (n = 1275, 69%). Graduates on entry to medical school (n = 585, 59%) were significantly less likely to pass than non-graduates (n = 3335, 66%). However, when considering programme type, candidates that attended graduate entry programmes (n = 420, 65%) did as well as standard entry programme candidates (n = 3465, 66%) and did better compared to other programme types. Candidates who passed MRCP Part 1 at first sitting had significantly higher scores in EPM than those who did not [median 0.299 (IQR − 0.123, 1.00) vs median − 0.299 (IQR − 1.01, 0.299)]. Similarly, those who passed MRCP Part 1 at first sitting had significantly higher scores in SJT than those who did not [mean SJT equated 676.61 (sd 25.23) vs 668.83 (sd 25.46)].

### Multivariable analysis

The following variables were included as independent covariates:

Model 1: age, sex, ethnicity, parental occupation, school type, graduate status and course type. Last medical school attended was entered as a random effect.

Model 2: age, sex, ethnicity, parental occupation, school type, graduate status, course type, EPM score and SJT mean deviate score. Last medical school attended was entered as a random effect.

Table 2 presents the findings of the mixed effects logistic regression models.

**Table 2:** Mixed effects logistic regression models assessing the relationship between passing first sitting of MRCP Part 1 with socio-demographic variables and educational indicators (fixed effects) and last medical school (random effect)

Covariate	Model 1 (excluding EPM and SJT scores) N = 5365			Model 2 (including EPM and SJT scores) N = 1425		
	Odds ratio	95% confidence interval	*p*-value	Odds Ratio	95% confidence interval	*p*-value
Age (years)	0.873	0.845, 0.902	< **0.001**	0.911	0.851, 0.975	0.07
Sex (reference group = women) Men	1.606	1.422, 1.814	< **0.001**	1.620	1.243, 2.111	** < 0.001**
EPM score (not in Model 1)	NA			4.108	3.402, 4.961	< **0.001**
SJT score (not in Model 1)	NA			1.003	0.998, 1.008	0.315
Ethnicity [reference group = majority ethnic (white)] Minority ethnic	0.613	0.530, 0.708	< **0.001**	1.166	0.870, 1.562	0.303
Parental occupation (reference group = professional/managerial) Non managerial/professional	0.923	0.804, 1.060	0.257	1.055	0.792, 1.406	0.713
School (reference group = state) Fee-paying	0.994	0.868, 1.139	0.932	1.233	0.928, 1.637	0.148
Grad on entry (reference group = not grad) Graduate	1.054	0.802, 1.385	0.706	0.646	0.359, 1.162	0.144
Course (reference group = standard entry) Medicine with a gateway year Medicine with a preliminary year Graduate entry course	0.489 0.767 1.442	0.265, 0.904 0.329, 1.792 1.045, 1.990	**0.022** 0.541 **0.026**	0.142 3.643 1.782	0.03, 0.684 0.392, 33.9 0.917,3.463	0.015 0.256 0.088

Statistically significant differences are shown in bold

Model 1: The odds of passing MRCP Part 1 at first sitting were significantly lower as age increased (OR = 0.873, 95% CI = 0.845, 0.902) and for those from a minority ethnic background (OR = 0.613, 95% CI 0.530, 0.708), while it was greater for men (OR = 1.606, 95% CI = 1.422, 1.814) Students from a Gateway to Medicine programme had significant lower odds to pass MRCP Part 1 at first sitting than students from a standard entry programme (OR = 0.489, 95% CI = 0.265, 0.904) and those on a graduate entry programme had greater odds to pass at first sitting than those on a standard entry programme (OR = 1.442, 95% CI = 1.045, 1.990).

Model 2: The odds of passing MRCP Part 1 at first sitting were greater for candidates passing who were male (OR = 1.620, 95% CI = 1.243, 2.111) and who had higher EPM scores (OR = 4.108, CI = 3.402, 4.961).

## Discussion

### Main findings

Our multivariable analysis of linked data held in UKMED shows that the odds of passing MRCP Part 1 at first sitting were significantly greater for men and those on a graduate entry medical programme. The odds of failing were significantly greater with age, if the candidate was from a minority ethnic group, or on a Gateway to Medicine programme. When the multivariable analysis included the SJT and EPM scores (the latter being a measure of performance at medical school), used for selection into the next stage of medical training, the Foundation Programme, odds were still significantly greater for passing in men, as well as candidates with higher EPM scores, but not for candidates from a minority ethnic group, or on a Gateway to Medicine programme.

Our study included a wider range of medical programmes and considered a greater number of potentially influencing factors than have previous similar studies (Dewhurst et al., 2007; McManus et al., 2020). By including medical programme as a random effect, we were able to control for the potential influence of type of medical school in the context of socio-demographic and educational performance indicators. Our findings suggest that success at first sitting of MRCP Part 1 is influenced by underlying socio-demographic and educational factors other than medical school attended (see later for further discussion).

### Comparison with previous literature

Previous research has conflicting messages about differences by sex on the MRCP Part 1, with some finding no difference (Dewhurst et al., 2007) and other papers finding women performed better than men (McManus et al., 2013). In contrast, we found men did better than women. The reason for this may be related to cohort differences, or differences over time, in terms of more women medical students and the feminization of medical schools and the medical workforce in the UK (Jefferson et al., 2015; Watts, 2018).

We were interested to see that candidates who entered medicine via a gateway programme performed less well than their peers on the UK’s traditional five-year undergraduate entry programmes. Gateway programmes were established to diversify the UK medical student population. As per (Curtis & Smith, 2020), “It is important to recognise that the background context for some students on gateway courses, which prevented them from realising their academic potential before entering medical school, will continue to have a negative effect on their undergraduate studies” (p2-3). This reflects our earlier point: the impact of disadvantage persists.

We were also intrigued by the fact that those on a graduate entry programme performed better than their peers on traditional five-year undergraduate entry programmes. This may be related to maturity, different life experience and perspectives and/or differences in ability compared to those on standard 5-year programmes (e.g., Garrud & McManus, 2018). This merits further explanation.

At first glance our findings related to ethnicity are consistent with previous research on undergraduate and postgraduate examination performance in medicine in relation to ethnicity (Woolf et al., 2011). However, this effect was no longer present when we included the education performance indicators. There was a clear pattern, similar to that seen in other studies of postgraduate examination performance, that performance is stable over time (Ellis et al., 2021; McManus et al., 2013; Scrimgeour et al., 2018a). Taken at face value, this might suggest that ability, not ethnicity, is a key determinant of performance. However, it is important to consider the complexity of differential attainment. Disadvantage at group level sets in very early in society, usually pre-school, and persists throughout education and into adult life (Garcia & Weiss, 2015, Farquharson et al., 2022). Socio-economic factors such as ethnicity may impact which school you go to, which then may impact on your education performance indicators, and so on. As we and others have said before (Fyfe et al., 2022), differential attainment is a complex, systemic problem reflective of unequal norms and practices within broader society, evident throughout many different assessments and persistent over time.

### Strengths and limitations of the study

To our knowledge, this large cohort study is the first to assess the relationship between specific sociodemographic factors and MRCP Part 1 success after adjusting for measures of prior academic attainment in medical school and type of medical programme. We made an a priori choice to focus on candidates who pass on first sitting, to enable comparison with similar studies of postgraduate examination performance (McManus & Ludka, 2012; Scrimgeour et al., 2017).

Big data studies are inevitably limited by the data that are available. Group sizes enabled all covariates of interest to be entered into our models. However, the EPM and SJT scores were introduced over the course of the period of investigation which meant models including these variables had lower numbers. Moreover, the number of candidates who had completed a gateway or extended programme was relatively small. Our investigations span the performance of candidates who first sat the MRCP Part 1 between 2013 and 2019. Patterns of examination performance in relation to demographic and educational indicators may have altered during, and subsequently to, this time span (as seems to be the case in respect of our study) and earlier research examining associations between sex and ethnicity in respect of MRCP Part 1 performance (Dewhurst et al., 2007)). Our study contains only doctors whose primary medical qualification (PMQ) was from a UK medical school during 2012–14, most of whom also lived and were high-school-educated in the UK (so-called “home students”). We cannot comment on the association between sociodemographic variables and MRCP Part 1 performance in international medical graduates (e.g., Unwin et al., 2018). The parameters of our analysis meant we could not include 2012–14 graduates who did less than full time foundation programme training (LTFT). However, the number of LTFT Foundation Doctors was small at the time of data collection (data from the General Medical Council [GMC]: Less than full time by survey year. Available: https://reports.gmc-uk.org/analytics) so we are confident that the exclusion of this group did not impact significantly on the outcomes. Ethnicity was categorised within the dataset into White and non-White, to maximise the sample size and resultant statistical power for comparisons. While pragmatic, this approach may fail to recognise the diversity and intersectionality of identities and experiences within such broad groupings (Dewhurst et al., 2007; Fyfe et al., 2022).

### Implications for research, policy and practice

Differential attainment in the MRCP may be the result of the assessment itself, variation in learning and training experiences, or variation in broader opportunities outside work. The first seems unlikely: a recent study indicated that the MRCP Part 1 (written) questions are mostly fair (Hope et al., 2018). In terms of the second, only one group difference (sex) remained when prior performance was entered into the model, suggesting that, at least in those UK doctors passing MRCP Part 1 on first sitting, there is reasonable equity. However, this group necessarily have higher and less variable scores than their peers who resit MRCP Part 1. Further research is needed to see if the same patterns of performance are apparent in candidates who resit MRCP Part 1 and international medical graduates, or whether these groups have different patterns of performance.

As per the current study, most studies of DA in postgraduate medical examinations have been single specialty. It is critical to know if there are differences across specialties, as lessons may be learned from those specialities which have less evidence of DA. Thus, future research should be multi-specialty, examining if DA is pervasive across medicine or if patterns differ in different specialties. It is also critical to acknowledge intersectionality by examining multiple variables (e.g., gender, sex, sociodemographic background) rather than just looking at limited variables/personal characteristics.

## Conclusion

Our findings provide a starting point for understanding how the accumulation of social and educational disadvantage or privilege can impact performance on MRCP part 1. Stepping back, big quantitative data analysis can only highlight patterns and tends to do so on “easily collected” variables. Reducing the measure of being a good doctor to performance on formal examinations is at odds with the discourse of embracing difference within the profession (e.g., Health Education England, 2022). Indeed, limiting judgements to a simple measure of examination performance may exacerbate differences and reinforce the status quo. Instead, we should use outcomes on major examinations as a way of assessing how different medical schools support doctors from different educational and social backgrounds to reach their full potential.

## Data Availability

UK Medical Education Database (“UKMED”) [UKMEDP088] extract first generated on 14/03/2019 and then refreshed on 06/09/2021. Approved for publication on 30/09/2022. I am grateful to UKMED for the use of these data. However, UKMED bears no responsibility for their analysis or interpretation. The data includes information derived from that collected by the Higher Education Statistics Agency Limited (“HESA”) and provided to the GMC (“HESA Data”). Source: HESA Student Record 2002/2003 and 2016/2017 Copyright Higher Education Statistics Agency Limited. The Higher Education Statistics Agency Limited makes no warranty as to the accuracy of the HESA Data, cannot accept responsibility for any inferences or conclusions derived by third parties from data or other information supplied by it. The dataset is held in safe haven and only members of the research team, IC, KW, RV and BK had access to the data. Researchers wishing to re-analyse the data used for this study can apply for access to the same data via UKMED.
